# Role of TGF-β signaling in inherited and acquired myopathies

**DOI:** 10.1186/2044-5040-1-19

**Published:** 2011-05-04

**Authors:** Tyesha N Burks, Ronald D Cohn

**Affiliations:** 1 McKusick-Nathans Institute of Genetic Medicine, Johns Hopkins University School of Medicine, Baltimore, MD 21205, USA; 2 Department of Pediatrics and Neurology, Johns Hopkins University School of Medicine, Baltimore, MD 21205, USA

## Abstract

The transforming growth factor-beta (TGF-β) superfamily consists of a variety of cytokines expressed in many different cell types including skeletal muscle. Members of this superfamily that are of particular importance in skeletal muscle are TGF-β1, mitogen-activated protein kinases (MAPKs), and myostatin. These signaling molecules play important roles in skeletal muscle homeostasis and in a variety of inherited and acquired neuromuscular disorders. Expression of these molecules is linked to normal processes in skeletal muscle such as growth, differentiation, regeneration, and stress response. However, chronic elevation of TGF-β1, MAPKs, and myostatin is linked to various features of muscle pathology, including impaired regeneration and atrophy. In this review, we focus on the aberrant signaling of TGF-β in various disorders such as Marfan syndrome, muscular dystrophies, sarcopenia, and critical illness myopathy. We also discuss how the inhibition of several members of the TGF-β signaling pathway has been implicated in ameliorating disease phenotypes, opening up novel therapeutic avenues for a large group of neuromuscular disorders.

## Introduction

The transforming growth factor-beta (TGF-β) superfamily plays a crucial role in normal physiology and pathogenesis in a number of tissues. It is important to emphasize that downstream effects of this signaling cascade are often tissue-specific, thereby dictating which target genes will be activated in response to the transduction signal. Given its multifaceted effects in different tissues, deregulation of TGF-β signaling cascades can lead to a multitude of developmental defects and/or disease [1]. Several members of the TGF-β family have been shown to play important roles in regulating muscle growth and atrophy. The most extensively characterized ligands, in terms of the effects on skeletal muscle, are TGF-β1, mitogen-activated protein kinases (MAPKs), and myostatin. In this review, we focus on these signaling molecules in normal homeostasis and pathological conditions affecting skeletal muscle and describe the therapeutic avenues that have recently been explored to target the TGF-β signaling cascade.

### Overview of the TGF-β superfamily signaling cascade

The TGF-β superfamily of cytokines consists of a variety of signaling molecules including isoforms of TGF-β (1 to 3), bone morphogenic proteins (BMPs 1 to 20), growth and differentiation factors (GDFs), activins (A and B), inhibins (A and B), nodal, leftys (1 and 2), and Mullerian inhibiting substance [1]. They are generally divided into two branches defined by the utilization of receptor Smads (R-Smads): the TGF-β branch, consisting of TGF-β, activin, Nodal, and myostatin (GDF-8), signals through R-Smads 2 and 3 and the BMP branch, consisting of BMPs and other GDFs, signals through R-Smads 1, 5 and 8. This superfamily is known to be involved in embryonic development, adult tissue homeostasis, and disease pathogenesis. Specifically, it has been shown to control proliferation, differentiation, apoptosis, migration, extracellular matrix (ECM) remodeling, immune functions, and tumor invasion/metastasis [2].

**Figure 1:**
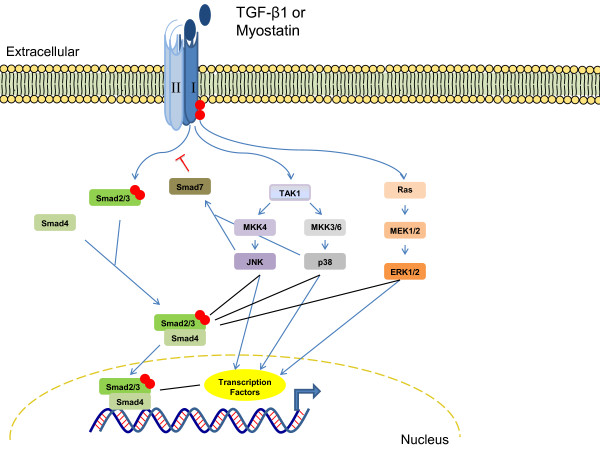
**Crosstalk between the canonical and non-canonical transforming growth factor-beta1 (TGF-β1) and myostatin pathways**. Once the TGF-β1 or myostatin ligands bind to the appropriate type I and type II receptors, cross-phosphorylation of the type I receptor occurs, leading to the phosphorylation of downstream effectors. In the canonical pathway, the type I receptor phosphorylates Smad2/3, which then binds to Smad4 and translocates into the nucleus to act as transcription factors. In the non-canonical pathway, the type I receptor phosphorylates proteins that are involved in the activation of the mitogen-activated protein kinases (MAPKs). Activated MAPKs can then regulate transcription factors and/or the Smad proteins through direct interactions or via downstream proteins.

TGF-β1 is synthesized as a precursor that is cleaved intracellularly into an inactive complex consisting of the mature TGF-β1 non-covalently bound to the portion of the precursor peptide termed the latency-associated peptide (LAP) [3]. This inactive TGF-β1-LAP complex forms a larger complex with latent transforming growth factor-binding proteins (LTBPs), which directly bind and release TGF-β1 from the ECM. Specifically, LTBP-4 sequesters and regulates the availability of TGF-β1 to bind with its receptor [4]. Cleavage of TGF-β1 from the latent complex is achieved through the action of proteases such as plasmin, thrombin, plasma transglutaminases, or endoglycosylases, or through the physical interaction of LAPs with other proteins [3]. Activation occurs extracellularly [3], and once TGF-β1 is released, it is able to interact with and complex its type I (usually TβR-II) and type II (usually activin receptor-like kinase (ALK) 5) receptors. The constitutively active type II receptor phosphorylates and activates the type I receptor, which in turn directly phosphorylates Smad2 and/or Smad3 (which are recruited by adaptor proteins) to initiate signal transduction through the canonical cascades [5]. Once R-Smad has been phosphorylated, it forms a complex with the common mediator Smad (co-Smad), Smad4, which translocates to the nucleus, where it directly binds defined elements on the DNA [2]. Adding to the regulation are the inhibitory Smads 6 and 7. Smad7 is involved in both branches and competes with R-Smads for interaction with the type I receptor, whereas Smad6 only participates in the BMP pathway and competes with Smad4 for binding to Smad1 [5] (Figure 1).

TGF-β1 can also signal via induction of non-canonical pathways including MAPK. The MAPK family consists of isoforms of extracellular signal-regulated kinases (ERKs) (1 and 2), c-Jun N-terminal kinase (JNKs) (1to 3), and p38 (α, β, γ and δ). The mechanisms of MAPK activation by TGF-β1 and the subsequent biological consequences are cell-type-specific [6]. Generally in the non-Smad pathway, the type I receptor associates with the adaptor proteins, Shc and tumor necrosis factor receptor-associated factor (TRAF) 6, for the activation of Ras and TGF-β-activated kinase (TAK) 1 and subsequently, the ERK and p38/JNK pathways, respectively [7]. However, MAPK may also modulate TGF-β1-induced Smad signals and phosphorylate Smad proteins independent of TGF-β1, providing evidence for crosstalk between canonical and non-canonical TGF-β pathways [6,7] (Figure 1).

Myostatin (MSTN), predominantly expressed in skeletal muscle, also signals through the TGF-β branch [8]. It is synthesized as a precursor protein that undergoes processing by furin proteases to generate a propeptide. After proteolytic processing, however, the biologically active MSTN remains bound non-covalently to the propeptide, and in this complex, the propeptide maintains its inactive, latent state [9,10]. MSTN also seems to be regulated extracellularly by other binding proteins: follistatin [9,11], follistatin-related gene (FLRG) protein [12], and growth and differentiation factor-associated serum protein (GASP) 1 [13]. When not bound to its propeptide or binding proteins, active MSTN is able to signal to target cells by binding to the activin type II receptors, ActRIIA or ActRIIB [14,15]. The activation of the type I receptor (usually ALK5 and to a lesser extent ALK4) leads to the phosphorylation of R-Smads 2 and 3 [15]. More recently, it has been shown that MSTN is also able to induce the activation of the MAPK signaling pathway in Smad-dependent and -independent mechanisms [16-18], and to inhibit the Akt/TORC1/p70S6K signaling pathway [19] (Figure 1). For a more extensive summary of MSTN, see [20].

### Physiological role of TGF-β signaling in skeletal muscle

TGF-β1 is expressed during myogenesis, and its spatial and temporal expression in the developing connective tissue is correlated with the fiber-type composition of the surrounding myotubes. Myotubes formed before the expression of TGF-β1 develop into slow fibers, whereas fast fibers form when myoblasts are adjacent to connective tissue expressing TGF-β1 [21]. TGF-β1 has been shown to inhibit the differentiation of fetal myoblasts but does not affect embryonic myoblasts [22]. In mature adult muscle, TGF-β negatively affects skeletal muscle regeneration by inhibiting satellite cell proliferation, myofiber fusion, and expression of some muscle-specific genes [23]. Furthermore, TGF-β1 induces the transformation of myogenic cells into fibrotic cells after injury [24].

Not much is known about the role of the different MAPKs in embryogenesis [25]; although, they have been shown to play a role in myogenesis and regeneration. p38 is speculated to regulate regeneration through the activation of p21, a cyclin-dependent inhibitor that causes irreversible withdrawal from the cell cycle (necessary for the differentiation of myoblasts) and through interactions with Pax7, myogenic regulatory factors, and myocyte enhancer factors [26,27]. JNK is proposed to inhibit myogenesis [28], and ERK may have multiple roles: preventing the initiation of myogenesis [29], enhancing myoblast proliferation during the acute stages, and repressing muscle-specific gene expression and myoblast differentiation, if expression is sustained [30]. Generally, in mature muscle, MAPKs mediate the transduction of diverse external stress stimuli into intracellular signals that regulate adaptive cellular responses such as proliferation, differentiation, self-renewal, and survival in diseased and healthy states [2,31]. For example, MAPK levels are modulated during exercise and aging as a stress response [31,32].

Myostatin is expressed in developing skeletal muscle throughout embryogenesis and has been shown to be a negative regulator of adult skeletal muscle mass by acting on different mechanisms [20]. Genetic studies in mice, cattle, sheep, dogs, chickens, and humans have all shown that myostatin normally functions to limit muscle mass [33-40]. In mice, targeted ablation of the *Mstn *gene causes a doubling of skeletal muscle mass throughout the body, as a result of a combination of muscle fiber hyperplasia and hypertrophy [33]. Moreover, postnatal inhibition of myostatin signaling through the delivery of propeptides, neutralizing antibodies, antisense RNA, inhibitory proteins, and soluble ActRIIB has been shown to induce significant muscle growth when administered to mice of different ages, demonstrating the importance of this signaling pathway in regulating muscle homeostasis [10,14,41-50].

### TGF-β signaling and skeletal-muscle repair

**Figure 2:**
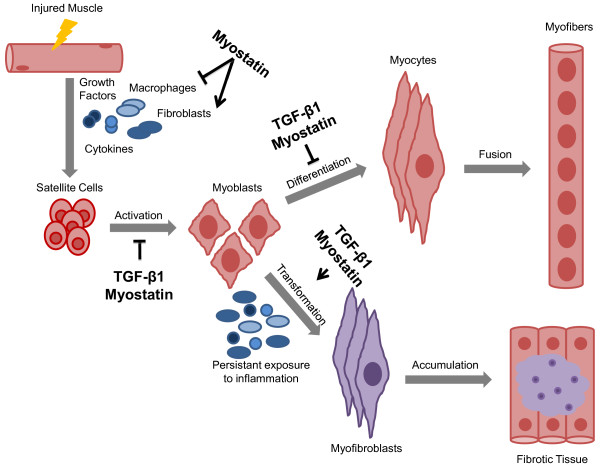
**Regulated and dysregulated muscle regeneration**. In regulated muscle regeneration, a transient inflammatory response occurs upon injury, which includes the chemotaxis of growth factors, cytokines, macrophages, and fibroblasts. This is followed by the activation and proliferation of satellite cells. Once activated, myoblasts differentiate into myocytes, and then fuse together to form myofibers, which exhibit central nuclei. This process is primarily orchestrated by the expression of the myogenic regulatory factors. In dysregulated muscle regeneration, there is a persistent inflammatory response and overexpression of proteins such as transforming growth factor-beta1 (TGF-β1) and myostatin, which promote the formation of fibrotic tissue to replace damaged myofibers.

After skeletal muscle injury, a well-coordinated repair process occurs. This process includes the release of growth factors and cytokines and the migration and proliferation of macrophages and fibroblasts that increase the production of ECM components; these components are degraded as normal regeneration occurs. The inflammatory response serves to clear myofiber debris and modulate regeneration. The formation of new myofibers begins with the activation of satellite cells, followed by proliferation, differentiation, and fusion of myocytes [51] (Figure 2).

TGF-β1, a potent regulator of tissue wound healing and fibrosis, is physiologically upregulated in regenerating skeletal muscle after injury and exercise and is thought to participate in a transient inflammatory response to muscle damage [51,52]. Persistent exposure to the inflammatory response leads to an altered ECM and increased levels of growth factors and cytokines, including TGF-β1, which contribute to the formation of fibrotic tissue [51,52]. Therefore, TGF-β1 is one of the major factors promoting the transformation of myoblasts into fibrotic tissue after injury. Furthermore, increased levels of TGF-β1 inhibit satellite cell activation and impair myocyte differentiation [23,53] (Figure 2). Interestingly, reducing the levels of TGF-β1 in various physiological and pathological conditions associated with muscle homeostasis and regeneration has proven to be beneficial for several myopathic conditions [54-70] (Table 1).

**Table 1:** Comprehensive overview of studies using agents to blunt transforming growth factor (TGF)-β signaling

Compound	Mechanism of action	Clinical condition	Model organism	Phenotypic findings	Ref
**FDA-approved medications**					

Losartan	AT1^a ^receptor antagonist (mostly used for hypertension, cardiomyopathies)	MFS^b^	*Fbn*^1C1039G/+ ^mice	Improved muscle architecture, function and regeneration	[54]
		
		DMD^c^	*mdx *mice	Improved skeletal, diaphragmatic and cardiac muscle architecture, function and regeneration	[54,55]
		
		Muscle Injury	Young^e ^mice	Decreased fibrosis and improved regeneration	[56]

Suramin	TGF-β1 receptor antagonist (anti-parasitic, anti-neoplasic)	DMD	*mdx *mice	Decreased fibrosis and prevented decrease in grip strength	[57]
		
		Muscle injury	Adult^f ^mice	Decreased fibrosis, improved regeneration and function	[58-60]

**Anti-fibrotic agents**					

Decorin	Binds to TGF-β1 ligands	Muscle injury	Young mice	Decreased fibrosis, improved regeneration and functional recovery	[61]
		
		DMD	*mdx *mice	Decreased collagen type I levels in diaphragm	[62]

γ-Interferon	Induces Smad7 expression	Muscle injury	Young mice	Decreased fibrosis, improved regeneration and functional recovery	[63]

Pirfenidone	TGF-β1 antagonist	DMD	*mdx *mice	Improved cardiac function, minor alterations on the development of fibrosis, and no improvement in diaphragmatic function	[64,65]

Halofuginone	Inhibits TGF-β-dependent phosphorylation of Smad3	DMD	*mdx mice*	Decreased fibrosis and improved function of the heart,diaphragm and limb muscles	[66,67]
		
		CMD^d^	*dy^2J^/dy^2J^*	Decreased fibrosis and improved functional performance but did not improve strength	[68]

**TGF-β neutralizing antibody**					

Neutralizes TGF-β (1 and/or 2) ligands		MFS	*Fbn*^1C1039G/+ ^mice	Prevented muscle atrophy and improved regeneration	[54]
		
		DMD	*mdx *mice	Decreased fibrosis and improved regeneration	[54,69]
		
		Sarcopenia	Aged^g ^mice	Failed to improve regeneration	[70]

**TGF-β receptor kinase inhibitor**					

Decoy receptor composed of extracellular portion of TGF-β receptor II		Sarcopenia	Aged mice	Improved regeneration after direct intramuscular injection	[70]

^a^Angiotensin II type 1 receptor.

^b^Marfan syndrome.

^c^Duchenne muscular dystrophy.

^d^Congenital muscular dystrophy.

^e^Age ≤ 3 months.

^f^Age 3-15 months.

^g^Age ≥ 15 months.

Myostatin also impairs skeletal muscle regeneration. It is proposed to hinder the chemotaxis of macrophages and myoblasts [71], while simultaneously activating and attracting fibroblasts to the site of injury. Once fibroblasts are within the environment of the injured muscle, they express MSTN and differentiate into myofibroblasts, a process that in turn accelerates the deposition of collagen and connective tissue, ultimately promoting the formation of tissue fibrosis [72,73]. Furthermore, myostatin inhibits the activation, differentiation, and self-renewal of satellite cells [71,74,75] and the expression of the muscle regulatory factors crucial for the regeneration and differentiation process of myofibers [76,77] (Figure 2). Inhibiting MSTN in various myopathic conditions has yielded mixed results, depending on the disease model and mechanism of inhibition [43,44,48,50,78-100] (Table 2).

**Table 2:** Comprehensive overview of studies using post-natal inhibition of myostatin

Disease	Model organism	Phenotypic findings	Ref
**Neutralizing antibody: binds to active myostatin and prevents receptor binding**			

DMD^a^	*mdx *mice	Improved regeneration and function, induced hypertrophy, decreased degeneration (diaphragm) and fibrosis	[78,79]

LGMD2C^b^	*sgcg*^-/- ^mice	Improved function, induced hypertrophy but no histopathological improvement	[80]

LGMD2F	*sgcd*^-/- ^mice	Increased muscle mass, regeneration (young) and fibrosis (aged)	[81]

ALS^c^	SOD1^G93A^ mice and rats	Delayed onset of muscle atrophy and functional decline without extending survival	[82]

Sarcopenia	Aged^f ^mice	Prevented loss of body weight, muscle mass and function, and decline in physical activity, reduced apoptosis, no change in fibrosis	[44,83]

Disuse atrophy	Adult^g ^mice	Partially protected against but did not prevent atrophy	[99]

**ActRIIB-Fc**^d^**: soluble, decoy receptor binding active myostatin **			

DMD	*mdx *mice	Increased body weight and function, induced hypertrophy	[84,100]

LGMD1C	CAV-3^P104L ^mice	Induced muscle hypertrophy	[85]

SMA^e^	SMAΔ7 mice	Modestly increased muscle weight and strength, decreased survival	[86]

ALS	SOD1^G93A ^mice	Delayed onset of disease but did not extend survival, reduced weakness after onset	[87]

Cachexia	Lewis-lung carcinoma	Protected against loss of body weight and muscle mass	[88]

Cachexia	Colon-26 carcinoma	Protected against or restored loss of body weight, muscle mass and grip strength, and increased survival	[88,89]

**MSTN Propeptide: binds to myostatin and prevents release of active form**			

DMD	*mdx *mice	Induced hypertrophy, increased strength, improved histopathological features of limb and diaphragm, decreased endurance, produced adverse effects on cardiomyopathy	[48,50,90]

LGMD2A	*Capn3*^-/- ^mice	Increased muscle mass and force, no improvement in histopathological features	[91]

LGMD2D	*sgca*^-/- ^mice	Insufficient delivery of vector resulted in no hypertrophy or any change in necrosis	[91]

Muscle Injury	Adult mice	Increased muscle mass, improved regeneration, decreased fibrosis	[92]

**Follistatin: inhibitory protein that binds to myostatin**			

SMA	SMAΔ7 mice	Improved muscle mass (during early stages of disease), motor function and extended survival	[93]

ALS	SOD1^G93A ^mice	Increased muscle mass (hyperplasia) and strength (not performance) but no survival extension	[94]

**HDAC Inhibitors: induce expression of follistatin**			

DMD	*mdx *mice	Induced hypertrophy, decreased fibrosis and necrosis, restored muscle architecture, increased strength and performance	[95]

LGMD2D	*sgca*^-/- ^mice	Induced hypertrophy and reduced fibrosis	[95]

Cachexia	Colon-26 carcinoma	Did not protect against loss of body weight, muscle mass or function	[88,96]

Muscle injury	Young^h ^mice	Improved regeneration	[97]

**MSTN peptide: dominant negative truncated myostatin peptide that binds ActRIIB**			

Sarcopenia	Aged mice	Improved grip strength and enhanced inflammatory response after injury	[98]

Muscle injury	Adult mice	Improved regeneration, decrease in necrosis	[98]

**Antisense RNA: binds myostatin messenger RNA and inactivates it**			

Cachexia	S-180 ascitic tumor	Increased muscle mass	[43]

^a^Duchenne muscular dystrophy.

^b^Limb-girdle muscular dystrophy.

^c^Amyotrophic lateral sclerosis.

^d^Activin type IIB receptor.

^e^Spinal muscular atrophy.

^f^Age≥ 15 months.

^g^Age 3-15 months.

^h^Age ≤ 3 months.

### Role of TGF-β signaling in disease pathogenesis of inherited myopathies

Dysregulation of TGF-β signaling has been implicated in various pathological conditions affecting skeletal muscle, both inherited and acquired [51]. Inherited conditions can be progressive, and therefore, there are unique phenotypic characteristics that may require different modes of intervention. Indeed, increased levels of TGF-β, MAPK, and/or MSTN have been associated with spinal muscular atrophy and Kennedy disease [101,102], and inhibition of MSTN improves familial amyotrophic lateral sclerosis (ALS) [82,87], but this review focuses on altered signaling in the pathogenesis of Marfan syndrome (MFS) and the muscular dystrophies.

MFS is an autosomal dominant systemic disorder of connective tissue, caused by mutations in *FBN1*, the gene encoding the ECM protein, fibrillin-1 [103]. A large subset of patients exhibit a significant decrease in muscle mass, often associated with hypotonia, particularly during early childhood, and experience a life-long inability to increase muscle mass despite physical exercise. Histological analyses of skeletal muscle from fibrillin-1-deficient mice and patients with MFS demonstrated a decrease in the number and size of myofibers, accompanied by an increase in fibrosis, fat deposition, and the number of split fibers. Further molecular analyses revealed that an increase in TGF-β signaling was indeed responsible for the abnormal muscle phenotype and the impaired ability to regenerate muscle in response to injury. Interestingly, when TGF-β signaling was blunted via treatment with a TGF-β neutralizing antibody or losartan, mice deficient in fibrillin-1 exhibited normal muscle architecture and regeneration capabilities [54].

'Muscular dystrophy' (MD) is a term used to describe a group of over 30 inherited disorders characterized by variable progressive muscle weakness and wasting [104,105]. Genetic mutations in genes encoding proteins spanning every subcellular aspect of the myofiber have been described [105]. There are currently no unifying hypotheses integrating all forms of MDs, but various lines of evidence suggest that repeated cycles of degeneration and regeneration may eventually impair the ability of satellite cells to repopulate damaged muscle [104]. Once muscle regeneration declines, there is often an accumulation of inflammation and fibrosis, which results in an abundance of growth factors and cytokines including TGF-β1, as stated above [51,62,106-108]. Similarly, the levels of decorin and biglycan, components of the ECM that interact with cytokines such as TGF-β1, as well as modulations of MAPK and myostatin signaling, are altered in various forms of muscular dystrophies including congenital MD, Emery-Dreifuss MD (EDMD) and Becker MD [109-115]. This review will elaborate on findings in Duchenne and limb-girdle MDs.

Duchenne muscular dystrophy (DMD) is an X-linked disorder characterized by a complete lack of dystrophin, which renders the myofiber membrane unstable. Inflammation is thought to precede the overexpression of TGF-β1 and actual muscle wasting [116,117]. Additionally, other factors including ECM components, immune system components, osteopontin, and fibrinogen are increased and have been linked to fibrosis in patients and animal models of DMD [52,108-110,116,118-120]. Furthermore, increased levels of the MAPKs ERK1/2, JNK1, and p38 have also been suggested to play a role in the pathogenesis of the skeletal and cardiac muscle phenotype in animal models of DMD [121-123]. Insight into the different factors contributing to the fibrosis accompanying DMD has led to various mechanisms to improve the phenotype observed in cardiac and skeletal (diaphragm and limb) muscles (Table 1 and 2) [52,54,64,124,125].

Limb-girdle muscular dystrophy (LGMD) describes a group of disorders primarily affecting the shoulder and pelvic girdle muscles, which have both autosomal dominant and recessive inheritance, and involve a variety of proteins including sarcoglycans, dysferlin, and caveolin [105]. Studies in a *Drosophila *model of LGMD with γ/δ-sarcoglycan deficiency have shown that partial reduction of the *Drosophila *genes homologous to Smads 2/3, Smads 1/5/7, and Smad4 improved muscle function, as shown by increased climbing ability of the flies. Similarly, reducing the levels of the homologue for Smads 2/3 and Smad4 improved the heart tube phenotype [125]. Not only has this research provided a novel animal model for studying dystrophic disease processes, but the results also indicate that targeting the R-Smads and co-Smad may be of therapeutic interest. Furthermore, genetic manipulation of LTBP-4, a latent TGF-β binding protein discussed above, affected the severity of a mouse model of sarcoglycan-deficient LGMD2C, providing evidence for an important genetic modifier of MD. An insertion in the LTBP4 gene reduced proteolysis and Smad signaling [4]. Thus, targeting the latent complex of TGF-β1 opens up yet another therapeutic avenue for inhibiting this cytokine in various conditions affecting skeletal muscle.

### Acquired myopathies implicating aberrant TGF-β signaling in disease progression

Alterations in the expression of the TGF-β signaling cascades have also been linked to acquired forms of myopathies. Muscle atrophy caused by hypoxia [126], microgravity exposure [127], starvation [128], acute daily psychological stress [129], various models of disuse [130-137], cancer [138,139], sporadic ALS [140], HIV [141], and glucocorticoid steroids [142] is associated with increased activation of MAPK, TGF-β1, and/or MSTN. However, in this paper, we focus on sarcopenia and critical illness myopathy (CIM).

Sarcopenia refers to the physiological age-related loss of skeletal muscle mass and function [143]. Several changes occur with age, including a decrease in myofiber size and number and diminished ability of satellite cells to activate and proliferate in response to injury, leading to impaired muscle remodeling [144,145]. The molecular mechanisms underlying sarcopenia are largely unknown. However, alterations in the canonical and non-canonical TGF-β signaling pathways have been shown to play a role in the pathogenesis of sarcopenia. Studies in elderly men have demonstrated an increase in MAPK at baseline, suggesting that aging skeletal muscle is functioning under 'stress-like' conditions at rest [32]. However, a different study conducted in mice and humans found an age-related decrease in ERK signaling in skeletal muscle and satellite cells, suggesting a contribution to the impaired regeneration [146]. Clearly, more in-depth studies are necessary to characterize the role of MAPK signaling in aging. Additionally, alterations in the canonical TGF-β pathway include an increase in circulating TGF-β1 levels and pSmad3, which contributes to the enrichment of connective tissue within the ECM, creating an environment that interferes with satellite cell activation and proliferation and subsequent remodeling [70,145]. Other studies have shown an upregulation of MSTN [147] and that inhibition of MSTN results in an increase in muscle mass, function, and regeneration in sarcopenic mice, suggesting an important role for this protein in the process of age-related loss of muscle mass [44,98] (Table 2).

CIM is characterized by generalized progressive muscle weakness and atrophy, occurring in critically ill patients who are hospitalized in the intensive care unit [148,149]. There are several factors thought to contribute to the loss of muscle mass in CIM, including immobilization, systemic inflammation, high dose steroids, and other toxins [148]. The precise molecular mechanisms underlying CIM are unknown [148]; however, constitutively active members of the canonical and non-canonical TGF-β signaling pathways may contribute to the muscle phenotype. In fact, atrophic fibers with apoptotic features express TGF-β ligands and receptors, p38, and downstream effectors of pJNK [149]. It is therefore tempting to speculate that TGF-β inhibition may slow or halt the progression of CIM.

### Therapeutic inhibition of TGF-β signaling

Aberrant TGF-β signaling has an important role in inherited and acquired myopathies. Therefore, research has been aimed at identifying compounds that can attenuate the increased signaling of TGF-β1, MSTN, and/or MAPK levels in order to improve disease phenotypes.

Several compounds have been shown to reduce the levels of TGF-β1 in myopathies. These include FDA-approved medications with other primary clinical uses, anti-fibrotic agents, TGF-β neutralizing antibodies, and TGF-β receptor blockers. Most yielded favorable results, but there are some conditions in which blunting TGF-β signaling was not beneficial. Table 1 provides a comprehensive overview of existing agents targeting TGF-β signaling in specific disease models.

Losartan is a widely studied, FDA-approved drug commonly used in the treatment of hypertension. Its ability to attenuate TGF-β signaling in chronic renal disease, cardiomyopathies, and MFS [54,150,151] made it an appealing molecule in the treatment of myopathies associated with increased TGF-β signaling. Long-term administration of losartan to dystrophin-negative *mdx *mice attenuated TGF-β signaling, decreased skeletal muscle fibrosis, and improved muscle regeneration and *in vitro *and *in vivo *function [54]. Furthermore, long-term administration of losartan, in conjunction with exercise, in *mdx *mice improved the cardiac muscle function and decreased fibrosis in the cardiac, diaphragm, and limb muscles but did not improve limb muscle function [55]. Other mechanisms of TGF-β inhibition have also yielded favorable results in the treatment of DMD and other conditions (Table 1).

Furthermore, recent experimental evidence has identified novel therapeutic targets in the TGF-β pathway. Molecules involved upstream (LTBP-4) and downstream (R-Smads and co-Smad) have been shown to modulate disease severity [4,125]. It is important to emphasize that LTBP-4 is a specific target for TGF-β1 [4], whereas Smad molecules incorporate a variety of different pathways, which could potentially lead to a number of adverse effects if they were therapeutically modified [125,152] (Figure 1). Moreover, osteopontin, an inflammatory regulator that also modulates TGF-β1, has recently been shown to be upregulated during muscle regeneration and in DMD [118,153-155]. Lack of osteopontin in *mdx *mice improved fibrotic tissue formation and muscle function, making osteopontin a potential therapeutic target [118].

In addition to altering TGF-β1 signaling, a number of compounds have been shown to inhibit myostatin signaling. Numerous studies breeding myostatin-null mice to several mouse models of inherited and acquired myopathies have shown various beneficial and non-beneficial effects [81,85,88,98,156-159]. Interestingly, several studies on myostatin-null mice alone have shown that despite an increase in muscle fiber size, there is no increase in specific force, which is probably due to a disturbance in mitochondrial metabolism [160,161]. Furthermore, myostatin-null mice have also been reported to have brittle tendons, which may contribute to the decrease in specific force [162]. However, it is important to emphasize that these studies were performed in mice with a complete lack of myostatin during the development of skeletal muscle. Thus, caution is needed when extrapolating the findings obtained from myostatin-null mice to the various compounds targeting myostatin signaling postnatally.

There are several pharmacological compounds that inhibit MSTN postnatally: MSTN propeptide, MSTN peptide, inhibitory proteins (follistatin), MSTN neutralizing antibodies, histone deacetylase inhibitors, and soluble ActRIIB. These techniques have been used in various disease models, and a detailed overview is presented in Table 2.

The soluble receptor, ActRIIB, is currently being used in multiple clinical trials and has been explored in various animal models including a model of DMD. It is important to note that targeting ActRIIB could lead to adverse side effects, because its expression is not limited to skeletal muscle [84] and because other members of the TGF-β superfamily besides myostatin bind to it [14]. Furthermore, myostatin can also signal through another receptor, ActRIIA, but with lower affinity [15].

Preclinical trials with soluble ActRIIB in *mdx *mice have shown that short-term (3 months) intraperitoneal administration increased skeletal muscle mass and *in vitro *function and caused a decrease in creatine kinase levels [100]. Adeno-associated virus (AAV)-mediated gene transfer of a soluble form of the extracellular domain of the ActRIIB to the liver provided similar results after 3.5 months, but no changes to cardiac muscle mass were seen [84]. However, long-term (11 months) myostatin inhibition using a recombinant AAV to overexpress myostatin propeptide in *mdx *mice did not reduce the amount of fibrosis in the diaphragm, but caused cardiac hypertrophy and impaired function in a dose-dependent manner [50]. These results indicate that all modes of myostatin inhibition may not be beneficial.

Similar to TGF-β1 and MSTN, perturbations of MAPK signaling have been documented in several myopathies, but not many studies exist examining the effects of inhibition on disease progression. Some evidence suggest that a reduction in JNK and ERK signaling might be beneficial in DMD [122] and cachexia [163], respectively, but further studies are needed to elaborate on these initial findings. Furthermore, a number of studies have shown that inhibition of the MAPK pathway is beneficial for the cardiomyopathic phenotype of various muscular dystrophies. Specifically, it has been shown that blunting ERK or JNK before and after onset of EDMD results in less cardiac fibrosis and an overall improved function [164-166]. Thus, this is a potential area of interest in designing future pharmacological compounds, because of the potential benefits and current lack of FDA-approved MAPK inhibitors [166].

## Conclusions

Increased activity of the TGF-β superfamily plays an important role in the pathogenesis of both inherited and acquired forms of neuromuscular disorders. These alterations cause an unfavorable environment for muscle regeneration and promote an increase in fibrotic tissue formation (Figure 2). Future studies will need to address the precise timeline of alterations in TGF-β signaling in various disease processes in order to establish the optimal therapeutic intervention. A number of drugs (Table 1; Table 2) are close to or currently in clinical trials. These and future clinical trials will need to establish the safety and efficacy of these drugs and address whether certain clinical conditions may benefit from a multi-targeted approach.

## Competing interests

The authors declare that they have no competing interests.

## Authors' contributions

TNB designed the study and drafted the manuscript. RDC drafted the manuscript. All authors read and approved the final manuscript.
